# The Heart Surgery Trailblazer's Enduring Legacy — in Memory of Professor Enio Buffolo

**DOI:** 10.21470/1678-9741-2025-0493

**Published:** 2026-05-06

**Authors:** Walter José Gomes

**Affiliations:** 1 Cardiovascular Surgery Discipline, Escola Paulista de Medicina, Universidade Federal de São Paulo, São Paulo, São Paulo, Brazil.

**Table:** 

Abbreviations, Acronyms & Symbols				
EPM	= Escola Paulista de Medicina		HSP	= Hospital São Paulo
OPCAB	= Off-pump coronary artery bypass surgery		UNIFESP	= Universidade Federal de São Paulo

"God grants everyone a star.

Some turn their star into a sun.

Others cannot even see it."

*Helena Kolody - Brazilian poet*

Heart surgery is the youngest offspring of the surgical domain. While brain, abdominal organs, and reconstructive surgery have been performed for millennia, the heart itself remained inviolable to surgeons' touch, considered as the seat of the soul and an insurmountable frontier, even for the most daring practitioners. The advent of cardiopulmonary bypass in 1953, merely 70+ years ago, enabled the intracavitary approach to the heart, profoundly changing this stalled picture and igniting a radical revolution that propelled cardiac surgery forward.

Thereafter, heart surgery thrived through groundbreaking advancements that dramatically reshaped the treatment of cardiac diseases. In just a few decades, a fresh generation of brilliant, gifted, and skilled pioneering surgeons pushed the boundaries of innovation. Their inventive techniques, coupled with novel technologies, fundamentally transformed the field. This epic endeavor places today these surgeons in a pantheon of almost mythical figures — both worldwide and, in parallel, in Brazil.

Professor Enio Buffolo has carved his own path through this constellation of talented, trailblazing minds and hands, actively building the field of heart surgery during its prime times. This personal tribute essay focuses on his journey through an academic lifetime at the university, specifically at the Hospital São Paulo (HSP), the teaching hospital of the Escola Paulista de Medicina (EPM) of the Universidade Federal de São Paulo (UNIFESP), where he graduated in 1965.

Professor Buffolo's vocation and formative career were shaped by distinguished and gifted mentors, including Professors Costabile Gallucci, Hugo Fellipozzi, and Vicente Forte, among others. Professor Gallucci served as the catalyst in establishing the chair of Thoracic Surgery at the HSP. A man of sensitivity, compassion, and a gregarious nature, he built a renowned surgical school and trained a team of young surgeons who became part of the elite of heart surgery in Brazil. His most well-known and quoted motto that embodies his philosophy was: “Each person who grows is a part of me that grows; therefore, I am growing”. He was ultimately succeeded in the chair of the Thorax Discipline by Professor Buffolo.

Professor Buffolo excelled beyond his predecessors and colleagues. A visionary, one-of-a-kind talent, he made outstanding, groundbreaking contributions, transforming the practice and field of cardiac surgery. He did not chase excellence in just one field but in several. Among his most notable achievements were the development of off-pump coronary artery bypass surgery (OPCAB), the introduction of disruptive techniques in reconstructive aortic disease surgery, and the establishment of transcatheter treatment for aortic valve stenosis.

OPCAB development was an outstanding and impressive achievement. The landmark 1996 publication of the initial OPCAB series in the *Annals of Thoracic Surgery*, a leading journal of cardiovascular surgery, demonstrating the feasibility of the surgical revascularization of the heart without the aid of cardiopulmonary bypass, with the heart beating, immediately galvanized the entire world, prompting surgeons globally to adopt the attractive new procedure. It achieved significant international impact and is considered one of the most cited Brazilian works in international medical literature^[1]^.

Developing OPCAB was once an unimaginable task. The procedure required suturing tiny coronary arteries to a graft while the heart was in motion, a challenge made even greater by the initial lack of today's supportive technological devices, such as stabilizers, positioners, intracoronary shunts, or short-acting intravenous beta-blockers. Only a generation of skilled, intrepid, and maverick surgeons could make this happen and achieve such remarkable outcomes.

However, developing OPCAB at that historical moment demanded not only skill but boldness and intellectual courage. Professor Buffolo challenged entrenched global paradigms, resisted skepticism, and pursued an idea many considered implausible. This capacity to defend an innovation against significant resistance — and ultimately to prove its value — became one of the most defining characteristics of his legacy.

Our institution became a Mecca for the peregrination of surgeons from around the world, eager to learn the new technique. This work elevated the HSP (EPM-UNIFESP) to the forefront of global medical innovation.

My own career was also directly shaped by this breakthrough advancement. Driven by the significant enthusiasm for the new technique among American and European surgeons, I accepted an invitation from the University of Bristol and the Bristol Heart Institute in the United Kingdom to develop further the OPCAB project, including the technique, safety, benefits, and core concepts. From 1997 to 1998, we developed clinical and operative trials research under the leadership of Professor Gianni Angelini. Our results provided the reassuring, evidence-based data necessary to validate the technique's safety, effectiveness, and applicability. During this period, we generated much of the primary evidence supporting the feasibility and benefits of OPCAB while actively promoting its worldwide adoption^[2,3]^.

To offer a glimpse into the excitement surrounding the new procedure, Professor Eulogio Martinez, then Head Professor of Cardiology, with his vivid personality, once compared the off- pump technique to changing a tire on a moving car.

The surgical team led by Professor Bufollo consisted of skilled, highly competent, and dedicated surgeons. I learned immensely from them, and their influence greatly enhanced my professional development. To acknowledge key individuals without indulging in oblivion, I would name Professors José Carlos Andrade, José Ernesto Succi, Luiz Eduardo Leão, Clotario Cuevas, and João Nelson Branco, as well as the tremendous expertise of the anesthetic team. Each played a significant role in shaping my training and surgical proficiency.

The atmosphere and intellectual ambience at the HSP and EPM during that time were vibrant and wonderful, a truly remarkable period that established the institution as one of the best academic programs in the country. Not only did cardiac surgery benefit, but all areas within EPM flourished. The combination of competence, hard work, and passion served as the key ingredients for success that ultimately propelled heart surgery advancements within our institution. Furthermore, we must acknowledge the subject of our work: the heart itself. The heart is unique, beautiful, and inspires passion; we invariably fall in love with it at first sight. This same feeling is shared by our students, who, during their time in the operating room, are typically thrilled and ecstatic at the vision of the beating heart.

In developing the techniques of reconstructive aortic surgery, Professor Buffolo partnered with his longtime friend Professor Domingo M. Braile, alongside Honorio Palma, Adalberto Camin, Nikolaus Geisthovel, and João Carlos Leal. Together, they assembled an early model of the aortic endoprosthesis — a device that revolutionized the treatment of aortic aneurysms and dissections. This partnership between two brilliant minds proved fruitful once again, laying the foundation for the transcatheter treatment of aortic valve stenosis. This involved the creation of stented valves inserted through the apex of the left ventricle, paving the way for further evolution to percutaneous transfemoral aortic valve implantation technology. These prostheses were developed and manufactured in Brazil by a local company, Braile Biomedica. Importantly, this partnership reflected Professor Buffolo's firm belief that Brazil should build its own medical technology rather than depend exclusively on imported devices. His advocacy for national biomedical innovation contributed to a more autonomous and inventive Brazilian cardiovascular industry^[4]^.

A commanding presence, sometimes a stern and austere figure, but one who nonetheless inspired discipline, dedication, and commitment in people working alongside him. Under his leadership and mentorship, dozens of surgeons were trained, many of whom later became also professors. With remarkable energy, he directed numerous operations each day, moving from one operating room to another and also from one hospital to another, often outworking teams of younger residents and fellows. We learned from his example, the discipline, commitment, resilience, and hard work that ultimately molded our lives and careers. In our routine these days, we used to wake up at 4 a.m. daily, arriving at the hospital before 5 a.m., preparing the patient, and starting the operation so it would be ready by 7 a.m. for his arrival. The first operation was usually concluded by lunchtime, then followed by a light meal before starting the afternoon case — usually the most complex one — leaving the hospital late at night, only to start the cycle over the next day. However, rather than discouraging or disheartening us, this demanding experience battle-hardened us all. It made us well-trained and resilient, fit to survive and succeed in this highly competitive field, which primarily demands physical stamina and emotional strength. Above all, we learned that we were not simply doing a job — we were doing something we loved.

Professor Buffolo was a methodical and confident surgeon — we learned from him and we apply daily in our surgical practice the teaching that surgical operations should be performed with a consistent, perfected routine rather than as an "adventure" where the technique is constantly modified, with steadiness and precision ensuring a higher standard of performance to achieve perfection. This teaching is also passed on to our residents every day in the operating room.

We lived through the romantic and fervent era of cardiac surgery, adhering strictly to the academic triad of patient care, teaching, and research. The aphorism “A smooth sea never made a skilled sailor” precisely defines our training. This commitment extended to weekend on-call duties in the intensive care unit to look after patients who had been operated on throughout the week. I recall my wife bringing our little son to the hospital on weekends so that he could see and know he had a Dad. Apparently, this was not detrimental or uninspiring; by the way, my son followed suit and became a heart surgeon as well. The comprehensive training program offered a unique academic experience, making it possible for us to develop master's and doctoral theses. From heart surgery and the daily care of very sick patients, we learned how tenuous the edge is between life and death and how our rigorous training and ensuing experience help us shift the pendulum of the patient's fate.

Taking advantage of his extensive relationships with other institutional leaders, his network within the university yielded fruitful collaborative work that propelled advancements in several correlated areas. One influential project involved a partnership with Professor José Carlos Prates from the Division of Descriptive Anatomy, Department of Morphology (Professor Prates at that time was regarded as one of the world's greatest anatomists). Our research focused on the surgical anatomy of the internal thoracic arteries; the findings regarding their ramification and vascularization laid the groundwork for the coronary artery bypass grafting's technical refinement of skeletonized harvesting of these conduits^[5]^. In collaboration with Professor Antonio Carlos Carvalho from the Cardiology Discipline, we produced the original description of the vasoplegic syndrome. Although initially discredited by the international community, this postoperative complication is now recognized as one of the commonest, deadliest, and costliest complications, prompting recent intense research for its understanding and optimized treatment following heart surgery^[6,7]^. Again, despite confronting widespread skepticism and a concept many regarded as questionable, subsequent evidence affirmed the accuracy of our observations and validated our early conclusions^[8]^.

Further collaborations were conducted with Professors Carl Peter von Dietrich and Helena Bonciani Nader — leading scientists from the Biochemistry Department who pioneered studies on the biological structure and function of proteoglycans, especially heparin and heparan sulfate. These studies placed particular emphasis on developing high-molecular-weight heparin for use in on-pump heart surgery and on understanding the role of these compounds in hemostasis after cardiac procedures^[9-11]^. Further, our pioneering work on the experimental use of videoendoscopy in minimally invasive coronary artery surgery involved collaboration with Professors Saul Goldenberg and Murched O. Taha from the Division of Surgical Techniques and Experimental Surgery, Department of Surgery^[12]^. Later in his career, his research focused on multifaceted areas like surgical alternatives for refractory heart failure and the emerging field of cell therapy in cardiology^[13]^.

Professor Buffolo also represented Brazilian cardiac surgery internationally. He participated in global meetings, presented innovations abroad, and helped establish Brazil as a respected contributor to cardiovascular science. His presence on the international stage strengthened the voice of Brazilian surgeons and elevated the country's visibility in the global surgical community.

Beyond his professional attributes, his charismatic personality bound people to him for their entire lives. Two such instances were Tarsila Tiengo, his nurse assistant who also served as a team manager, and Miyoko Omoto, the HSP's operative theater head nurse. Strong, tireless, and devoted, both women dedicated most of their lives to cardiac surgery and to Professor Buffolo. Having worked alongside them for decades, I can attest that they were two of the most loyal and devoted people I have ever met.

Upon his retirement from the university, I was selected to succeed him in the position of Head Professor, following a highly competitive process. While it is a challenging task, we strive to excel and leverage the institution to reach even higher standards. To achieve this, we keep moving forward and refining the OPCAB concept, with technical and operative advancements, and this is demonstrated by the addition of the anaortic strategies, which have further reduced perioperative complications and improved outcomes^[14]^.

The commitment to implement high-quality surgery for large volumes of public-system patients (from the Sistema Único de Saúde) within a public university hospital (the HSP) has been expanded. This was achieved by widely spreading the use of OPCAB, a technique particularly suited to resource-limited environments. The program was extended to other university- affiliated hospitals performing cardiac surgery, and dedicated courses for training new surgeons on the technique have been established. Ultimately, his innovations had a profound social impact, not only scientific significance.

Professor Enio Buffolo passed away peacefully on October 5th, 2025, at the age of 83, leaving a profound and enduring legacy in the Brazilian and worldwide cardiovascular surgery community. To wrap up this essay, I acknowledge that while a legacy doesn't die, it may be reshaped or reinterpreted over time. In these challenging times for cardiac surgery, may his powerful example of life and achievements inspire a new generation of heart surgeons to love what they do and continue building the standard of excellence in cardiac surgery for decades to come. Paraphrasing the song by Elis Regina, with her marvelous voice, "the new always comes" — may new generations of heart surgeons carry forward the essence of the passionate and ardent work developed by the trailblazers who built our field.

## Data Availability

The authors declare that the data supporting the findings of this study are available within the article.

## Artificial Intelligence Usage

The author declares that Grammarly was used to check for spelling, grammar, and plagiarism detection. DeepSeek was used to check sentence structure, conciseness, and tone. The content produced by the artificial intelligence tool was revised and edited by the author as necessary, and he takes full responsibility for the content to be published.
